# Association between acute mountain sickness (AMS) and age: a meta-analysis

**DOI:** 10.1186/s40779-018-0161-x

**Published:** 2018-05-11

**Authors:** Yu Wu, Chi Zhang, Yu Chen, Yong-Jun Luo

**Affiliations:** 1 Department of Military Medical Geography, Army Medical Service Training Base, Army Medical University, Chongqing, 400038 China; 2 Key Laboratory of High Altitude Environmental Medicine of PLA, Army Medical University, Chongqing, 400038 China; 3 Battalion 5 of Cadet Brigade, Army Medical University, Chongqing, 400038 China

**Keywords:** Meta-analysis, Age, Acute mountain sickness, Individual susceptibility

## Abstract

**Background:**

Acute mountain sickness (AMS) is a potentially lethal condition caused by acute hypoxia after ascending to altitudes higher than 2500 m in a short time. The main symptom of AMS is headache. Numerous risk factors of AMS have been examined, including gender, obesity, ascent rate, age and individual susceptibility. In previous studies, age was considered a predisposing factor for AMS. However, different opinions have been raised in recent years. To clarify the association between AMS and age, we conducted this meta-analysis.

**Methods:**

We obtained observational studies that explored risk factors for AMS by searching PubMed, Embase, China National Knowledge Internet (CNKI), the Wanfang database and CQVIP for articles published before March 2017. The studies included were required to provide the mean age and its standard deviation for subjects with and without AMS, the maximum altitude attained and the mode of ascent. The Lake Louse Score (LLS) or the Chinese AMS score (CAS) was used to judge the severity of AMS symptoms and incidence. Studies were pooled for the analysis by using a random effects model in RevMan 5.0. Meta-regression and subgroup analyses were conducted to identify sources of heterogeneity using Stata 14.2 and RevMan 5.0.

**Results:**

In total, 17 studies were included, and the overall number of subjects with and without AMS was 1810 and 3014, respectively. The age ranged from 10 to 76 years. Analysis of the 17 included studies showed that age was not associated with AMS (mean difference (MD) = 0.10; 95% CI: -0.38-0.58; *P* = 0.69).

**Conclusion:**

This meta-analysis suggests that there is no association between age and the risk of AMS. Race, age, and ascent mode are common sources of heterogeneity, which may provide an analytical orientation for future heterogeneity analyses.

## Background

Acute mountain sickness (AMS) is a syndrome with non-specific symptoms and is characterized by headache accompanied by other symptoms, including nausea, fatigue, dyspnea, insomnia, difficulty sleeping and dizziness [1–3]. AMS usually appears after 6 h after ascending to high altitude (higher than 2500 m) or ascending rapidly to a higher altitude, and it reaches a peak in 12–96 h [4]. The non-specific symptoms of AMS can become more serious after the first night at altitude and typically resolve spontaneously after 1 or 2 days, barring further ascent [5–7].

Numerous factors have been investigated in previous studies as potential risk factors for AMS, such as gender [8], smoking [9, 10], obesity [11, 12], ascent rate [6, 13–15], history of AMS [12, 16, 17], age [2, 5, 6, 18–22], training status [12, 23–25], sleep quality [26] and physical activity [13, 14, 27]. In addition, individual susceptibility was considered a vital risk factor for AMS [12, 16, 17, 28–30]. In particular, numerous studies have reported that age is a risk factor. However, the conclusions of previous studies are not consistent. Moraga et al. found that children were more sensitive to AMS than were adolescents or adults when traveling to high altitudes [19, 31]. Hackett et al. [2, 22, 32] demonstrated that younger trekkers were more susceptible to AMS. In contrast, Honigman et al. [12, 21] reported that older travelers were more sensitive than youth to AMS and that the risk of AMS increased with age. However, the association between age and the risk of AMS was not reported by Schneider et al. [6, 33].

Given the differences in research methodology and subjects in these previous studies, it is unclear whether age is related to AMS. To assess this problem and provide new methods for predicting the risk of AMS, we conducted a meta-analysis to clarify whether a conspicuous relationship exists between age and the risk of AMS. Thus, we explored heterogeneity factors through meta-regression and subgroup analyses.

## Methods

### Literature search strategy

Two reviewers (YW and CZ) carried out a comprehensive literature search in PubMed, Embase and Chinese Database. We used the following search terms as medical subject headings and free-text words: “acute mountain sickness”, “acute high-altitude disease”, “acute mountain illness”, “incidence”, “questionnaires”, “risk factors” and “age” as well as combinations of those using “OR” and “AND”. We also searched the literature using the keywords “acute mountain sickness”, “acute high-altitude disease”, “incidence”, “epidemiologic investigation”, “questionnaires” and “age factors” for studies in Chinese from the China national knowledge infrastructure (CNKI), the Wanfang Database and CQVIP. In addition, the studies listed in the references of the articles were reviewed. The retrieval was conducted in March 2017. No relevant studies were published in languages other than Chinese and English. The full texts of the studies that met our criteria were downloaded after primary selection by reading the titles and abstracts. We contacted the corresponding authors when full texts were not available. This study was approved by the ethical committee of the Third Military Medical University in China.

### Study selection

This meta-analysis was based on observational studies, including cohort, case-control and cross-sectional studies. Articles were selected if they met the following four criteria. 1) The study provided the total number of subjects and the average age and standard deviation for both the AMS and non-AMS groups, either directly or indirectly using the original data. 2) Subjects traveled from lower altitude to higher altitude in less than 2 weeks. 3) The AMS status of subjects was declared. 4) The full text was accessible or provided by the corresponding authors. The following exclusion criteria were employed. 1) The article was a review or reported results of a pooled analysis. 2) The research object was an animal. 3) The overall sample size was fewer than ten participants.

### Data extraction

The following items were collected from each selected study: the first author, publication year, journal, altitude, number of participants with and without AMS, and LLS or CAS with a cut-off value, average age and standard deviation of subjects with and without AMS, and ascent mode and duration of the ascent.

### Definition of AMS

This analysis only included studies that defined AMS using either the Lake Louse Score (LLS) or Chinese AMS score (CAS). The cut-off values for both scores were defined by the authors of each study (Table 1). Therefore, studies that did not provide either of these scores but used alternative diagnosis criteria were excluded from this analysis.

**Table 1:** Characteristics of the studies included in the meta-analysis

Study	Max altitude (m)	Male/Female (*n*)	Sample size (*n*)			LLS or CAS cut-off value	Age range (mean, year)	Age (year)		*P*	Subject origin	Ascent mode	Ascent duration
			AMS	Non-AMS	Sum			AMS	No AMS				
Hackett, 1976 [2]	4243	–	146	132	278	Arbitrary symptom score ≥ 2	18–71(33)	31.4 ± 9.7	35.2 ± 13.3	0.029	Britain, USA, France, Germany, Switzerland, Japan, Other	Plane, Trek	4d
Wagner, 2006 [37]	4419	266/93	120	239	359	LLS ≥ 3	10–76(39.2)	34.40 ± 12.81	41.71 ± 14.71	< 0.0001	USA	Trek	1–3 d
Mairer, 2009 [38]	3500	316/106	70	361	431	LLS ≥ 4	-- (37.4)	38.4 ± 11.1	37.2 ± 13.1	0.20	Austria	Trek	–
Mairer (a), 2010 [39]	3454	64/11	30	45	75	LLS ≥ 4	-- (34.7)	35.1 ± 10.7	34.5 ± 10.2	0.79	Austria	Trek	–
Mairer (b), 2010 [39]	3817	63/17	28	52	80	LLS ≥ 4	-- (36.8)	36.2 ± 12.2	38.1 ± 10.4	0.47	Austria	Trek	–
Karinen, 2012 [40]	5600	34/22	24	12	36	LLS ≥ 3	24–45(32)	33 ± 7	30 ± 5	> 0.05	Finland	Trek	7–17 d
Wei, 2012 [34]	4000	–	276	924	1200	CAS ≥ 5	20–50(35)	35.36 ± 19.54	35.80 ± 16.85	> 0.05	China	–	–
Mao, 2012 [35]	4768	135/87	71	213	284	LLS ≥ 3	18–69(40.5)	43.5 ± 13.5	37.8 ± 12.7	< 0.05	China	Train	1.5 h
You, 2012 [41]	4300	314/0	119	195	314	LLS > 4	- (20.2)	20.08 ± 1.62	20.25 ± 1.83	0.414	China	Bus	4 d
Harrison, 2013 [42]	3200	55/35	30	60	90	LLS ≥ 3	- (35.4)	33.8 ± 9.2	36.2 ± 9.4	> 0.05	USA	Plane	< 4 h
Song, 2014 [36]	3900	73/0	37	36	73	LLS ≥ 3	18–26(20.5)	20.72 ± 2.21	20.33 ± 2.05	> 0.05	China	Car	5 d exposure
Wu, 2015 [44]	3886	102/77	80	99	179	LLS ≥ 3	11–13(11.8)	11.8 ± 0.5	11.8 ± 0.4	NS	China	Bus Walk	6 h + 2 h
Bian, 2015 [45]	3700	150/	84	66	150	LLS ≥ 3	18–60(22.2)	22.77 ± 3.83	21.39 ± 2.38	0.011	China	Plane	2 h
Ren, 2015 [46]	4300	31/49	35	45	80	LLS > 3	20–50(38.4)	38.1 ± 11.8	38.6 ± 8.3	0.829	China	Plane, bus	6 h
Li (a), 2015 [47]	3700	752/0	461	291	752	LLS ≥ 3	18–45(22.9)	23.0 ± 3.9	22.8 ± 3.9	0.475	China	Plane	2.5 h
Li (b), 2015 [47]	4400	267/0	104	163	267	LLS ≥ 3	18–45(22.0)	22.2 ± 2.5	21.8 ± 2.3	0.162	China	Car	3 h
Yu, 2016 [48]	3700	176/0	95	81	176	LLS ≥ 3	- (23.0)	23.21 ± 3.96	22.78 ± 3.45	0.181	China	Plane	2 h

*AMS* Acute mountain sickness, *LLS* Lake Louise score, *CAS* The Chinese AMS score, *SD* Standard deviation. -: No data or not mentioned

### Statistical analyses

We analyzed continuous data by calculating the mean differences (MD) with 95% confidence intervals (CI). A DerSimonian-Laird random effects model was used to aggregate the data. The combined effect size was evaluated using the inverse variance method. Heterogeneity between studies was tested using the Cochrane and *I*^2^ statistics. Publication bias was assessed using funnel plots.

The heterogeneity of the included studies was determined using a *Q* test and quantified using the *I*^*2*^ statistic. The significance level was defined as 0.1. If heterogeneity was significant, i.e., *P* ≤ 0.1 or *I*^*2*^ ≥ 50%, then a random effects model was required. If *P* > 0.1 and *I*^*2*^ < 50%, then the included studies were not heterogeneous, and a fixed effects model was more appropriate. Meta-regression analyses, subgroup analyses and sensitivity analysis were used to identify sources of heterogeneity. Meta-analyses, subgroup analyses and sensitivity analysis were conducted using the RevMan 5.0 software (The Cochrane Collaboration). Meta-regression analyses were conducted with Stata 14.2 (Stata Corp, College Station, TX, USA).

## Results

### Selection of literature

**Fig. 1:**
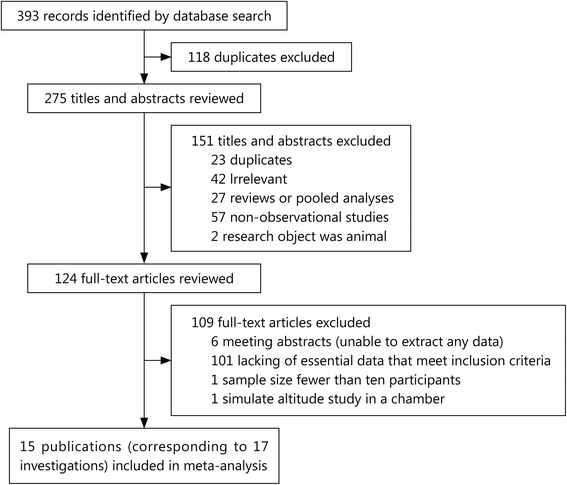
Flowchart of the literature review

As shown in the flow chart (Fig. 1), 15 publications corresponding to 17 investigations were identified for the analysis. In total, 393 articles were initially retrieved. Among the retrieved studies, 139 were in Chinese, and 254 were in English. After reading the titles and abstracts, 151 non-observational studies were excluded, leaving 124 articles to be evaluated in detail. In total, 17 studies were included in the meta-analysis, and 109 studies were excluded because they did not provide a LLS or CAS score or the age and standard deviation of the AMS and non-AMS groups.

### Characteristics of the included studies

Eighteen studies were included in the meta-analysis. Among them, 3 studies were in Chinese [34–36], and 14 studies were in English [2, 37–48]. The total number of subjects with and without AMS was 1810 and 3014, respectively. The mean study sample size was 270 participants (age range 10–76 years). Most of the included studies had a wide range of ages, which ensured the reliability of this study and avoided the effect of a variance in age in different studies. Table 1 lists the altitude and diagnosis of AMS with cut-off values.

### The relationship between age and AMS

**Fig. 2:**
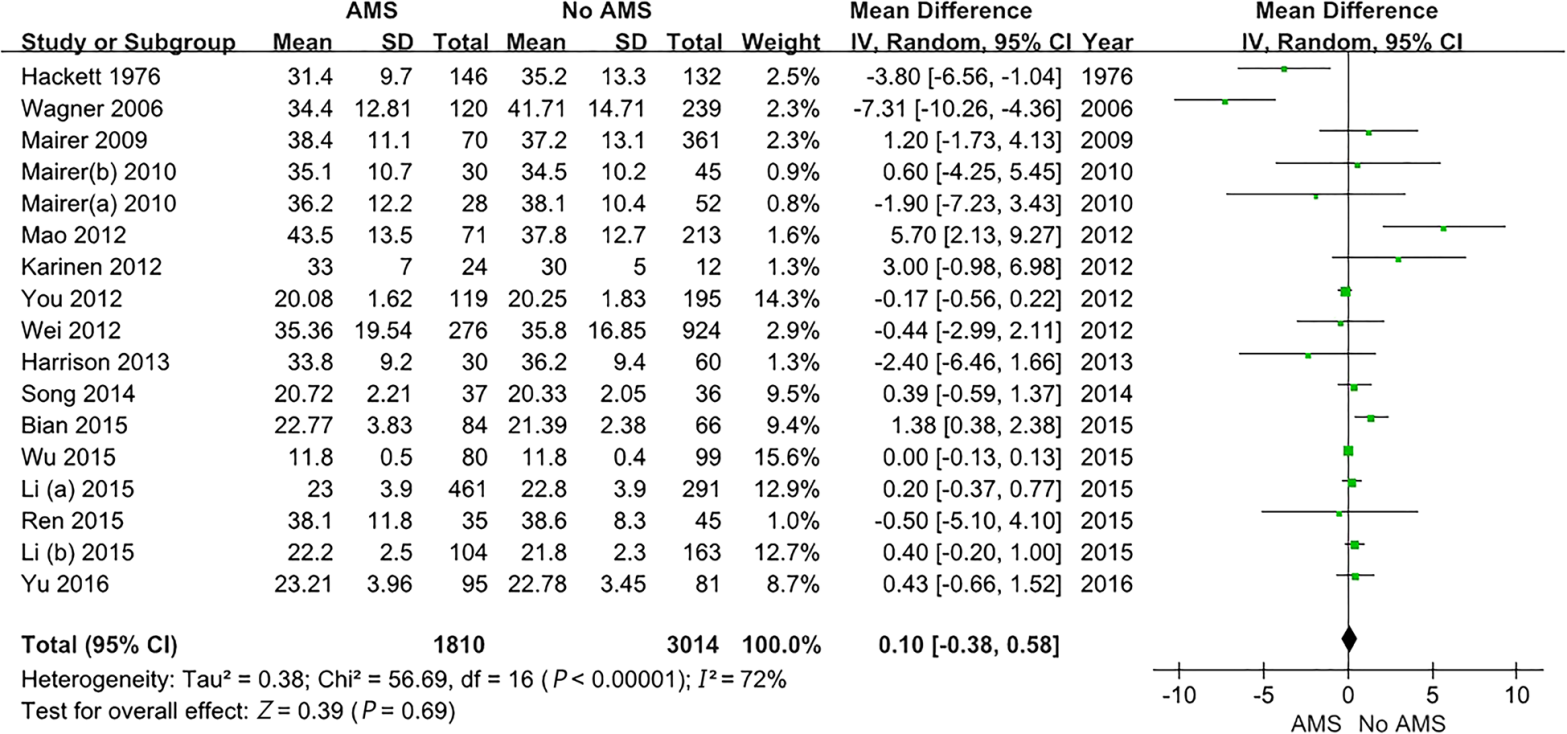
Forest plot. No statistically significant association was found between the risk of AMS and age. The 18 studies exhibited significant heterogeneity. AMS: Acute mountain sickness; SD: Standard deviation; CI: Confidence intervals; IV: Inverse variance

Significant heterogeneity was present among the 17 studies (*P* < 0.001, *I*^*2*^ = 72%); therefore, a random effects model was required. A meta-analysis of the 17 included studies suggested that no statistical significance was observed between the risk of AMS and age (MD = 0.10; 95% CI -0.38-0.58; *P* = 0.69). Detailed information is shown in Fig. 2.

#### Meta-regression analysis

The heterogeneity of the metadata was represented as *I*^2^ = 72%, df = 16, *P* < 0.001, which was considered highly heterogeneous. A meta-regression method was used to select the factors that might lead to heterogeneity (altitude, sample, race, and ascent mode), and 4 models were established:

$$ \mathrm{M}1=\mathrm{Altitude}+\mathrm{Sample}\ \mathrm{size}+\mathrm{Race} $$

$$ \mathrm{M}2=\mathrm{Altitude}+\mathrm{Sample}\ \mathrm{size}+\mathrm{Race}+\mathrm{Age} $$

$$ \mathrm{M}3=\mathrm{Altitude}+\mathrm{Sample}\ \mathrm{size}+\mathrm{Race}+\mathrm{Ascent}\ \mathrm{mode} $$

$$ \mathrm{M}4=\mathrm{Altitude}+\mathrm{Sample}\ \mathrm{size}+\mathrm{Race}+\mathrm{Age}+\mathrm{Ascent}\ \mathrm{mode} $$

In the meta-regression analysis, race (*P =* 0.002, *P* = 0, *P* = 0.039, *P* = 0.003, respectively) and age (*P* = 0.003, *P* = 0.001, respectively) reached statistical significance as heterogeneous elements. Ascent mode (*P* = 0.658, *P* = 0.05, respectively) was noted as a possible heterogeneous factor. Detailed information is presented in Table 2.

**Table 2:** Meta-regression results of 17 studies

Heterogeneous factors	Coefficient				Standard error				*Z*				*P* value			
	M1	M2	M3	M4	M1	M2	M3	M4	M1	M2	M3	M4	M1	M2	M3	M4
Altitude	0.0001	−0.0007	0.0001	0.0004	0.0003	0.0004	0.0005	0.0005	− 0.16	− 0.74	0.16	0.79	0.872	0.082	0.872	0.427
Sample size	0	−0.0014	0.0001	−0.0015	0.0005	0.0007	0.0005	0.0007	−0.04	−2.12	0.05	−2.27	0.968	0.034	0.963	0.023
Race	2.1531	3.4527	2.0841	3.4422	0.6841	0.8154	0.7018	0.8154	3.15	4.23	2.97	4.22	0.002	0	0.003	0
Age	–	0.0662	–	0.0785	–	0.0226	–	0.0240	–	2.93	–	3.27	–	0.003	–	0.001
Ascend mode	–	–	−0.1131	−0.4124	–	–	0.2558	0.2717	–	–	−0.44	−1.52	–	–	0.658	0.05
Constant	−1.8892	−1.2281	−2.3158	−2.6617	1.5536	1.5699	1.8291	1.8321	−1.22	−0.78	− 1.27	− 1.45	0.224	0.434	0.205	0.146

Race assignment: 1 means Asian, 0 means non-Asian; Ascend mode assignment: 1 means < 1 days to reach; 0 means ≥1 days to reach; *AMS* Acute mountain sickness. -: No data

#### Subgroup analyses

Subgroup analyses on race, age, and ascent mode were performed. In the age group, the 17 studies were divided into 3 groups using the thresholds of 18 and 30 years. No significant difference (*P* > 0.05) was noted. The Asian racial group, the 18 to 30-year-old age group, and the plane ascent modes exhibited significantly reduced heterogeneity (*I*^2^ = 57%, *I*^2^ = 48%, *I*^2^ = 35%, respectively) but still exhibited moderate heterogeneity. The specific results are presented in Table 3.

**Table 3:** Subgroup analysis results of 17 studies

Subgroup	Grouping criteria	No. of Studies	AMS/Non-AMS (*n*)	*I*^2^ (%)	IV (95%CI)	*P* value
Race	Non- sian	7	448/901	77	−1.65(−4.51,1.21)	0.26
	Asian	10	1362/2113	57	0.27(− 0.07,0.84)	0.12
Age (year)	< 18	1	80/99	–	0.00(−0.13,0.13)	1.00
	18–30	6	900/832	48	0.31(−0.08,0.70)	0.12
	≥30	10	830/2083	79	−0.64(−3.08,1.78)	0.60
Ascent mode	Trek	6	418/841	81	−1.51(−4.83,1.82)	0.37
	Car or Bus	6	716/575	61	0.14(−0.26,0.54)	0.50
	Plane	5	676/1598	35	0.49(−0.20,1.18)	0.16
Overall		17	1810/3014	72	0.10(−0.38,0.58)	0.69

*AMS* Acute mountain sickness; Age group criteria: Average age (mean). *IV* Inverse variance, *CI* Confidence intervals

-: No data

#### Sensitivity analyses

After eliminating the group with the most samples, the meta-analysis demonstrated that the MD was 0.11 (95%CI -0.38-0.60), suggesting no significant difference in age (*P* = 0.66) between the AMS and non-AMS groups. After eliminating the data group with the least samples, the meta-analysis revealed an MD of 0.06 (95% CI -0.42-0.54), suggesting no significant difference in age (*P* = 0.81) between the AMS and non-AMS groups. The results were the same when the MD values and their 95% CI were combined. Therefore, the sensitivity analysis revealed no bias from sample size.

After eliminating the research objects individually, the meta-analysis suggested that significant heterogeneity remained (*P* < 0.05, *I*^2^ > 50%) among the studies, and the combined results had no significant effects (*P* > 0.05). The sensitivity analyses did not identify the source of heterogeneity, and the meta-analysis results are robust.

### Publication bias

**Fig. 3:**
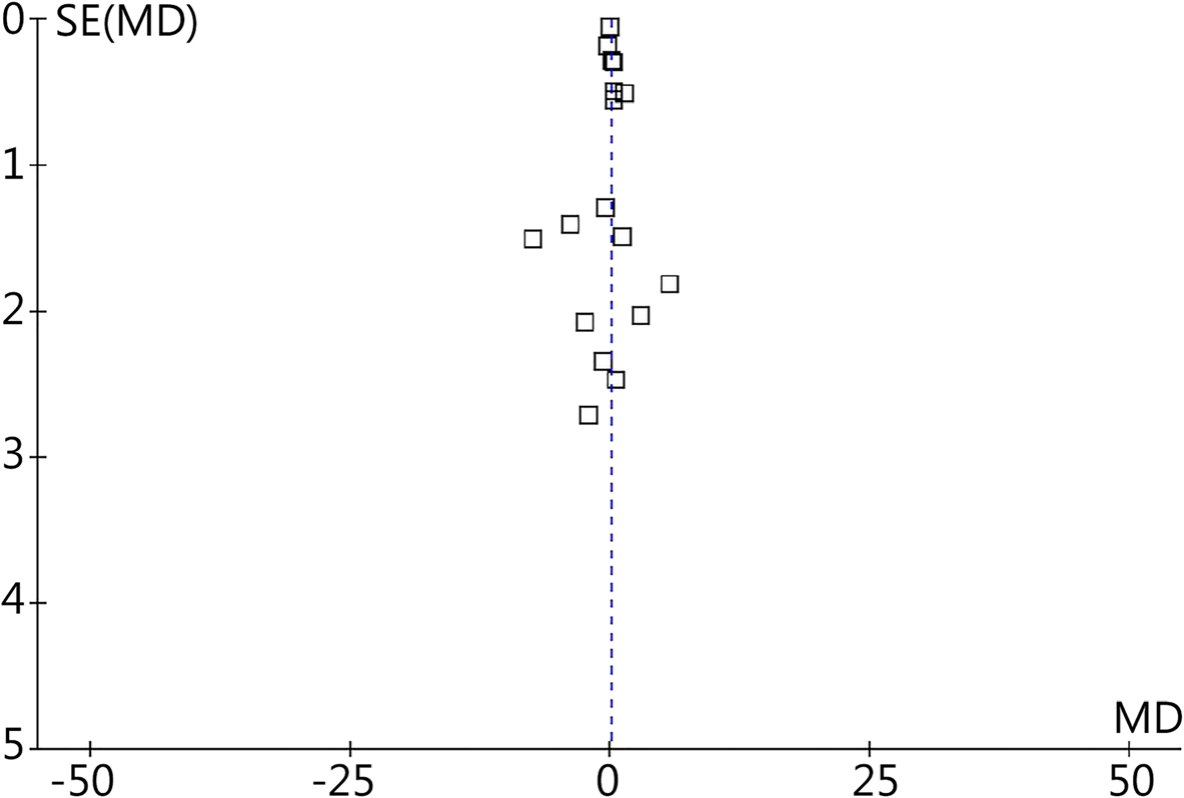
Funnel plot of all included studies. SE: Standard error; MD: Mean difference

Publication bias was measured using a funnel plot (Fig. 3). No asymmetry was observed. Thus, we determined that there was no significant publication bias among the 17 included studies.

## Discussion

This meta-analysis explored the relationship between age and the risk of AMS. After excluding the studies which did not meet our inclusion criteria, 17 studies were included in this meta-analysis, which included 1810 AMS and 3014 no-AMS patients. The results of this meta-analysis suggest that there is no correlation between AMS and age (MD = 0.10; 95% CI -0.38-0.58; *P* = 0.69).

Numerous studies explored the impact of age on the risk of AMS. However, these studies reported different results. Tang et al. [49] suggested that the elderly were more sensitive to AMS, whereas Hackett et al. [2, 22, 32] reported opposite findings, suggesting that older age is protective against AMS. In addition, some studies indicated that age has no relationship with AMS. The results of our meta-analysis indicate that there is no correlation between age and AMS. The following reasons may explain the results: 1) Many of the previous studies were conducted immediately upon arrival at a destination or 24 h later. AMS typically occurs 6–12 h after exposure to hypobaric hypoxia [50], and AMS symptoms gradually resolve after 1 or 2 days, barring further ascent or with proper rest [7]. Results that are obtained too early or too late are unable to detect an accurate association between age and the risk of AMS. 2) Among previous studies, the diagnostic criteria of AMS were inconsistent, which may have led to different results. In addition, diet control has not been properly performed in previous studies. Excessive water intake contributes to fluid retention and leads to intracranial hypertension at high altitudes, which may enhance the risk of AMS [43]. 3) Subjects in numerous previous studies came from different altitudes, which could impact the result. The subjects should be divided into several groups based on the altitude where they lived before conducting the studies. Thus, the conclusions gained from these results would be more convincing. 4) In fact, numerous factors affect AMS, including altitude, gender, ascent speed and methods, drug prevention, personal health, and individual susceptibility. However, numerous studies have not controlled for these other factors, and the interaction of various factors can affect a study’s results.

Hackett, Honigman and Gaillard [2, 12, 22] reported that the elderly were less likely to develop AMS. One hypothesis of the mechanism of AMS is brain swelling [51]. They theorized that brain size decreased with age, which increased the cranial compliance and caused a reduction in the risk of AMS [37, 52], leaving more intracranial space to accommodate brain tissue swelling without critical increases in intracranial pressure upon shifts of cerebrospinal fluid (CSF) downward into the spinal column. However, the brain size in AMS was also determined by baseline size, genetic predisposition and individual response to hypoxia [5]. Additionally, activity levels after arriving at high altitude were not controlled for in many previous studies. Younger individuals tend to have high activity levels compared with the elderly. Thus, younger people are more susceptible to AMS because movement boosts oxygen consumption in the brain and decreases oxygen saturation in brain tissue [33]. Hypoxia increases the release of free oxygen radicals and calcium overload, which increases vascular permeability and leads to brain swelling. In addition, older trekkers and climbers have more experience and better protection experience compared with young people, i.e., the avoidance of drug use and control of ascent rate. Finally, the small number of elderly participants caused a non-uniform age distribution in subjects, which could cause a deviation in the results. To eliminate the heterogeneity of the analysis, the population considered in this study was divided based on the ages of 18 and 30 years in the age subgroup analysis, but the results were not significantly different (*P* > 0.05).

Furthermore, Tang et al. [49] reported that the elderly were more likely to develop AMS due to poor sleep quality. Sleep quality decreases with age, and low-quality sleep may enhance the risk of AMS [49, 53]. However, sleep quality is affected by numerous factors, such as chronic pain, psychological problems, obesity and changes in sleeping conditions [54–56]. Additionally, previous studies in the elderly may have had unknown comorbidities or unreported illnesses that made them more sensitive to AMS [12, 33]. Harrison et al. stated that the development of AMS was associated with an increase in VEGF (vascular endothelial growth factor): Hypoxia increases VEGF in the serum, and VEGF increases vascular permeability, leading to brain swelling and the development of AMS [42]. However, there is currently no evidence that VEGF level was related to age.

Despite the strengths presented above, there are still several limitations to this study. First, because age is a basic demographic attribute, not all published studies report the age of the included subjects. Therefore, we could only include 17 studies in which age was properly described. Second, we only included studies that provided either an LLS or CAS score, which dramatically reduced the number of potentially eligible studies for this analysis. Some publications used AMS diagnosis from clinical data and did not provide AMS or CAS scores. Moreover, a random effects model was employed to ensure a conservative conclusion. Finally, in the subgroup analysis, some subgroups included only 1 paper given the limited number of included studies. Future research should increase the age data discussing the relationship between AMS and different age groups (such as children, young and elderly). Further studies should employ standardized diagnostic criteria and explore the effects of other contributors, such as gender, fitness and ascent rate.

## Conclusions

In summary, we performed a meta-analysis of selected studies reporting LLS or CAS for AMS to explore the association between age and the risk of AMS via heterogeneity factors through meta-regression and subgroup analysis. We found no association between the risk of AMS and age.

## Abbreviations

AMS: Acute mountain sickness
CAS: The Chinese AMS score
CI: Confidence intervals
CNKI: China National Knowledge Internet
IV: Inverse variance
LLS: Lake Louise score
LLSS: the Lake Louise scoring system
MD: mean difference
SE: Standard error
VEGF: Vascular endothelial growth factor

## References

[1] Singh I, Khanna P, Srivastava M, Lal M, Roy SB, Subramanyam C (1969). Acute mountain sickness. N Engl J Med.

[2] Hackett PH, Rennie D, Levine HD (1976). The incidence, importance, and prophylaxis of acute mountain sickness. Lancet.

[3] Heath D, Williams DR. High-altitude medicine and pathology. Oxford University Press. 1995.

[4] Zhang YB. High Altitude Disease. Qinghai People's Press. 1982.

[5] Roach RC, Hackett PH (2001). Frontiers of hypoxia research: acute mountain sickness. J Exp Biol.

[6] Schneider M, Bernasch D, Weymann J, Holle R, Bartsch P (2002). Acute mountain sickness: influence of susceptibility, preexposure, and ascent rate. Med Sci Sports Exerc.

[7] Bärtsch P, Swenson ER (2013). Acute high-altitude illnesses. N Engl J Med.

[8] Imray C, Wright A, Subudhi A, Roach R (2010). Acute mountain sickness: pathophysiology, prevention, and treatment. Prog Cardiovasc Dis.

[9] Wu TY, Ding SQ, Liu JL, Jia JH, Chai ZC, Zhao JZ (2012). Smoking, acute mountain sickness and altitude acclimatisation: a cohort study. Thorax.

[10] Vinnikov D, Blanc PD, Steinmaus C (2016). Is smoking a predictor for acute mountain sickness? Findings from a meta-analysis. Nicotine Tob Res.

[11] Hirata K, Masuyama S, Saito A (1989). Obesity as risk factor for acute mountain sickness. Lancet.

[12] Honigman B, Theis MK, Koziol-McLain J, Roach R, Yip R, Houston C (1993). Acute mountain sickness in a general tourist population at moderate altitudes. Ann Intern Med.

[13] Hackett PH, Roach RC (2001). High-altitude illness. N Engl J Med.

[14] Broome JR, Stoneham MD, Beeley JM, Milledge JS, Hughes AS (1994). High altitude headache: treatment with ibuprofen. Aviat Space Environ Med.

[15] Hackett PH, Rennie D (1978). Avoiding mountain sickness. Lancet.

[16] Forster P (1984). Reproducibility of individual response to exposure to high altitude. Br Med J (Clin Res Ed).

[17] Robinson SM, King AB, Aoki V (1971). Acute mountain sickness: reproducibility of its severity and duration in an individual. Aerosp Med.

[18] Ross RT (1985). The random nature of cerebral mountain sickness. Lancet.

[19] Bloch J, Duplain H, Rimoldi SF, Stuber T, Kriemler S, Allemann Y (2009). Prevalence and time course of acute mountain sickness in older children and adolescents after rapid ascent to 3450 meters. Pediatrics.

[20] Moraga FA, Pedreros CP, Rodríguez CE (2008). Acute mountain sickness in children and their parents after rapid ascent to 3500 m (Putre, Chile). Wilderness Environ Med..

[21] Rexhaj E, Garcin S, Rimoldi SF, Duplain H, Stuber T, Allemann Y (2011). Reproducibility of acute mountain sickness in children and adults: a prospective study. Pediatrics.

[22] Gaillard S, Dellasanta P, Loutan L, Kayser B (2004). Awareness, prevalence, medication use, and risk factors of acute mountain sickness in tourists trekking around the Annapurnas in Nepal: a 12-year follow-up. High Alt Med Biol..

[23] Hansen JE, Harris CW, Evans WO (1967). Influence of elevation of origin, rate of ascent and a physical conditioning program on symptoms of acute mountain sickness. Mil Med.

[24] Kayser B (1991). Acute mountain sickness in western tourists around the Thorong pass (5400 m) in Nepal. J Wilderness Med.

[25] Gupta JS, Joseph NT, Malhotra MS (1978). Physical fitness status and adaptation to high altitude. Indian J Med Res.

[26] Ziaee V, Yunesian M, Ahmadinejad Z, Halabchi F, Kordi R, Alizadeh R (2003). Acute mountain sickness in Iranian trekkers around mount Damavand (5671m) in Iran. Wilderness Environ Med..

[27] Roach RC, Maes D, Sandoval D, Robergs RA, Icenogle M, Hinghofer-Szalkay H (2000). Exercise exacerbates acute mountain sickness at simulated high altitude. J Appl Physiol (1985).

[28] Richalet JP, Kéromès A, Dersch B, Corizzi F, Mehdioui H, Pophillat B (1988). Caractéristiques physiologiques des alpinistes de haute altitude. Sci Sports.

[29] Zhou F, Wang F, Li F, Yuan J, Zeng H, Wei Q (2005). Association of hsp70-2 and hsp-hom gene polymorphisms with risk of acute high-altitude illness in a Chinese population. Cell Stress Chaperones.

[30] Jiang CZ, Li FZ, He MA, Sun SY, Zhang SY, Liao R (2005). Glutathione S-transferase M1, T1 genotypes and the risk of mountain sickness. Zhonghua Lao Dong Wei Sheng Zhi Ye Bing Za Zhi.

[31] Moraga FA, Osorio JD, Vargas ME (2002). Acute mountain sickness in tourists with children at Lake Chungará (4400 m) in northern Chile. Wilderness Environ Med.

[32] Roach RC, Houston CS, Honigman B, Nicholas RA, Yaron M, Grissom CK (1995). How well do older persons tolerate moderate altitude?. West J Med.

[33] Wu TY, Ding SQ, Liu JL, Jia JH, Chai ZC, Dai RC (2012). Who are more at risk for acute mountain sickness: a prospective study in Qinghai-Tibet railroad construction workers on Mt. Tanggula Chin Med J (Engl).

[34] Wei CM, Luo YH, Zhang Z (2012). Analysis of risk factors related to acute high altitude disease. Qinghai Med J.

[35] Mao YN, Zhang ZY, Wu QL, Qi SG, Yang L, Wu TY (2012). Incidence and risk factors of acute mountain sickness among Qinghai-Tibet railroad passengers. J High Alt Med.

[36] Song P, Qin J, Gao XB, Zhang JH, Yu J, Chen GZ (2014). Effect of acute high altitude exposure on lung functions and relationship between lung function and AMS. Mil Med Sci.

[37] Wagner DR, Fargo JD, Parker D, Tatsugawa K, Young TA (2006). Variables contributing to acute mountain sickness on the summit of Mt Whitney. Wilderness Environ Med..

[38] Mairer K, Wille M, Bucher T, Burtscher M (2009). Prevalence of acute mountain sickness in the eastern alps. High Alt Med Biol.

[39] Mairer K, Wille M, Burtscher M (2010). The prevalence of and risk factors for acute mountain sickness in the eastern and western alps. High Alt Med Biol..

[40] Karinen HM, Uusitalo A, Vähä-Ypyä H, Kähönen M, Peltonen JE, Stein PK (2012). Heart rate variability changes at 2400 m altitude predicts acute mountain sickness on further ascent at 3000-4300 m altitudes. Front Physiol.

[41] You H, Li X, Pei T, Huang Q, Liu F, Gao Y (2012). Predictive value of basal exhaled nitric oxide and carbon monoxide for acute mountain sickness. Wilderness Environ Med..

[42] Harrison MF, Anderson P, Miller A, O'Malley K, Richert M, Johnson J, et al. Physiological variables associated with the development of acute mountain sickness at the south pole. BMJ Open. 2013; 3(7): pii: e003064. 10.1136/bmjopen-2013-003064 PMC3717461 23869103

[43] Gatterer H, Wille M, Faulhaber M, Lukaski H, Melmer A, Ebenbichler C (2013). Association between body water status and acute mountain sickness. PLoS One.

[44] Wu SH, Lin YC, Weng YM, Chiu YH, Li WC, Wang SH (2015). The impact of physical fitness and body mass index in children on the development of acute mountain sickness: a prospective observational study. BMC Pediatr.

[45] Bian SZ, Jin J, Zhang JH, Li QN, Yu J, Yu SY (2015). Principal component analysis and risk factors for acute mountain sickness upon acute exposure at 3700 m. PLoS One.

[46] Ren XW, Zhang QY, Wang H, Hong H, Qiao HY, Man CY (2015). The relationship between baseline exhaled nitric oxide levels and acute mountain sickness. Am J Med Sci.

[47] Li M, Zhang JH, Zhao GX, Bian SZ, Gao XB, Liu X (2015). A specific objective supplemental factor in evaluating acute mountain sickness: ΔHR in combination with SaO_2_. Mil Med Res.

[48] Yu J, Zeng Y, Chen G, Bian S, Qiu Y, Liu X (2016). Analysis of high-altitude syndrome and the underlying gene polymorphisms associated with acute mountain sickness after a rapid ascent to high-altitude. Sci Rep.

[49] Tang XG, Zhang JH, Qin J, Gao XB, Li QN, Yu J (2014). Age as a risk factor for acute mountain sickness upon rapid ascent to 3,700 m among young adult Chinese men. Clin Interv Aging.

[50] Basnyat B, Murdoch DR (2003). High-altitude illness. Lancet.

[51] Loeppky JA, Icenogle MV, Maes D, Riboni K, Hinghofer-Szalkay H, Roach RC (2005). Early fluid retention and severe acute mountain sickness. J Appl Physiol (1985).

[52] Kallenberg K, Bailey DM, Christ S, Mohr A, Roukens R, Menold E (2007). Magnetic resonance imaging evidence of cytotoxic cerebral edema in acute mountain sickness. J Cereb Blood Flow Metab.

[53] Liu X, Uchiyama M, Kim K, Okawa M, Shibui K, Kudo Y (2000). Sleep loss and daytime sleepiness in the general adult population of Japan. Psychiatry Res.

[54] Kim SH, Lee DH, Yoon KB, An JR, Yoon DM (2015). Factors associated with increased risk for clinical insomnia in patients with chronic neck pain. Pain Physician.

[55] Palm A, Janson C, Lindberg E (2015). The impact of obesity and weight gain on development of sleep problems in a population-based sample. Sleep Med.

[56] Blank M, Zhang J, Lamers F, Taylor AD, Hickie IB, Merikangas KR (2015). Health correlates of insomnia symptoms and comorbid mental disorders in a nationally representative sample of US adolescents. Sleep.

